# Efficient Generation of Myostatin Mutations in Pigs Using the CRISPR/Cas9 System

**DOI:** 10.1038/srep16623

**Published:** 2015-11-13

**Authors:** Kankan Wang, Hongsheng Ouyang, Zicong Xie, Chaogang Yao, Nannan Guo, Mengjing Li, Huping Jiao, Daxin Pang

**Affiliations:** 1 Jilin Provincial Key Laboratory of Animal Embryo Engineering, College of Animal Sciences, Jilin University, Changchun, Jilin Province, People’s Republic of China

## Abstract

Genetically modified pigs are increasingly used for biomedical and agricultural applications. The efficient CRISPR/Cas9 gene editing system holds great promise for the generation of gene-targeting pigs without selection marker genes. In this study, we aimed to disrupt the porcine myostatin (*MSTN*) gene, which functions as a negative regulator of muscle growth. The transfection efficiency of porcine fetal fibroblasts (PFFs) was improved to facilitate the targeting of Cas9/gRNA. We also demonstrated that Cas9/gRNA can induce non-homologous end-joining (NHEJ), long fragment deletions/inversions and homology-directed repair (HDR) at the *MSTN* locus of PFFs. Single-cell *MSTN* knockout colonies were used to generate cloned pigs via somatic cell nuclear transfer (SCNT), which resulted in 8 marker-gene-free cloned pigs with biallelic mutations. Some of the piglets showed obvious intermuscular grooves and enlarged tongues, which are characteristic of the double muscling (DM) phenotype. The protein level of *MSTN* was decreased in the mutant cloned pigs compared with the wild-type controls, and the mRNA levels of *MSTN* and related signaling pathway factors were also analyzed. Finally, we carefully assessed off-target mutations in the cloned pigs. The gene editing platform used in this study can efficiently generate genetically modified pigs with biological safety.

The development of custom endonuclease techniques promotes the generation of genetically modified animals, which can be used for exploring gene function and generating animal models of human genetic diseases. ZFN, TALEN and CRISPR/Cas9 can introduce DNA double-stranded breaks at specific sites^1^,^2^,^3^, which are then repaired via non-homologous end-joining (NHEJ) or homologous recombination. Typically, the NHEJ repair process causes gene mutations with small deletions or insertions in the double-stranded break regions^4^. Among these three endonuclease techniques, CRISPR/Cas9 has emerged as a more powerful tool due to the resultant high efficiency^5^,^6^ and rapid assembly^7^. Many genetically modified animals, such as zebrafish^8^, *Drosophila*^9^, mice^10^ and monkeys^11^, have been generated using the CRISPR/Cas9 system.

As a member of the transforming growth factor-β superfamily, Myostatin (*MSTN*) acts as a negative regulator of muscle growth and holds great promise for improving livestock growth performance. *MSTN* knockout (KO) mice show a much greater muscle mass than wild-type mice^12^, and some cattle breeds that harbor natural mutations in *MSTN* also exhibit greater muscle mass^13^. Previous studies^14^,^15^,^16^ showed that the porcine *MSTN* gene experienced relatively different selective pressure than bovinae and caprinae *MSTN* during evolution. It is possible that *MSTN* presents different expression patterns or even plays a distinct role in pigs. Pigs are among the most important livestock in the world, and pork is consumed in particularly large quantities in China. Hence, the generation of *MSTN* knockout pigs is of great significance for investigating the effects on muscle development and meat performance.

In previous studies, gene-targeted pigs have been generated either through cytoplasmic injection of Cas9 mRNA and sgRNA into zygotes^17^,^18^ or via somatic cell nuclear transfer (SCNT) using targeted fibroblasts selected with antibiotics^19^. However, both of these methods have disadvantages. Co-injection of Cas9 mRNA and sgRNA into zygotes often results in chimeric animals with multiple genotypes, and it is time-consuming and costly to perform further breeding in pigs. Genetically modified pigs derived from fibroblasts selected with drugs often carry antibiotic resistance genes, and they are increasingly giving rise to concerns regarding biological safety.

In the present study, we improved the transfection efficiency of porcine fetal fibroblasts (PFFs) to approximately 90%, thus improving the mutation efficiency of targeting vectors. We also demonstrated CRISPR/Cas9 system-mediated gene editing at the *MSTN* locus of porcine fetal fibroblasts, including mutations arising through NHEJ, long fragment deletions and inversions, due to the co-transfection of two CRISPR/Cas9 targeting vectors and single-stranded DNA (ssDNA)-mediated homologous recombination. Finally, the Cas9/gRNA-modified fibroblasts without selection marker genes were used to generate gene-targeted pigs via somatic cell nuclear transfer, which resulted in 8 cloned piglets with biallelic mutations in *MSTN*.

## Results

### Optimizing transfection to improve the efficiency of gene targeting in porcine primary fibroblasts

**Figure 1:**
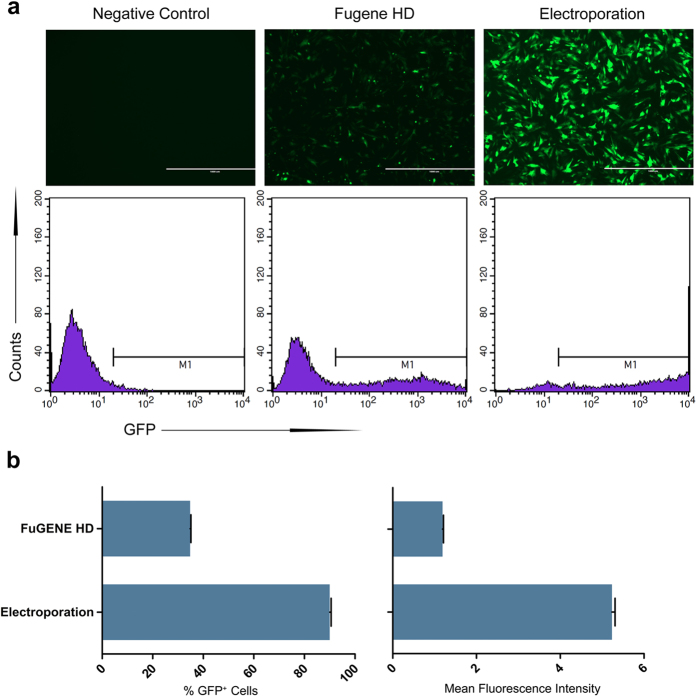
Optimization of PFF transfection. (**a**) The pEGFP-N1 expression plasmid was transfected into PFFs using the indicated methods. GFP fluorescence was analyzed via fluorescence microscopy and FACS at 24 h post-transfection. (**b)** Percent of GFP-expressing cells and mean fluorescence intensity of GFP-expressing cells.n = 3. Graphs show the mean ± S.E.M.

The low transfection efficiency of primary cells has always been a challenge in large animal transgene research. Several studies^20^,^21^,^22^ have focused on this problem, and many transfection strategies, including lipid-based delivery, electroporation and nucleofection, have been applied to porcine primary fibroblasts. To further investigate transfection efficiency, we introduced pEGFP-N1 into porcine fetal fibroblasts using electroporation and FuGENE HD. According to an electroporation study^22^, properly increasing the number of pulses contributes to a higher transfection efficiency. However, it can also lead to a higher cell death rate. In this study, we increased the amounts of cells and plasmids while also increasing the number of pulses, thus allowing a sufficient number of cells to survive after transfection. Moreover, we used Opti-MEM as the transfection medium, instead of expensive and complex electroporation buffers, thereby simplifying the transfection process. EGFP-positive cells were analyzed 24 h after transfection through fluorescent microscopy and fluorescence-activated cell sorting (FACS) (Fig. 1a). Following electroporation, nearly 90% of the survived cells displayed EGFP green fluorescence, while the FuGENE HD rate was approximately 35%. In addition, the mean fluorescence intensity of EGFP-positive cells transfected via electroporation was much higher than that obtained using FuGENE HD (Fig. 1b). To determine whether the optimized electroporation method could facilitate sgRNA-Cas9-mediated gene targeting, a specific CRISPR/Cas9 vector targeting the *EIF4G1*locus was transfected into PFFs. Sequencing chromatograms revealed that the electroporation group exhibited more obvious multi-peaks around the targeting site, which indicated a higher targeting efficiency (Supplementary Fig. 1). The subsequent TA cloning result further confirmed that the electroporation group presented a much higher mutation efficiency than FuGENE HD group (Supplementary Table 1). These results suggest that our optimized electroporation protocol enhanced gene targeting dramatically compared with the traditional delivery strategy.

### CRISPR/Cas9-mediated gene editing in PFF cells

**Figure 2:**
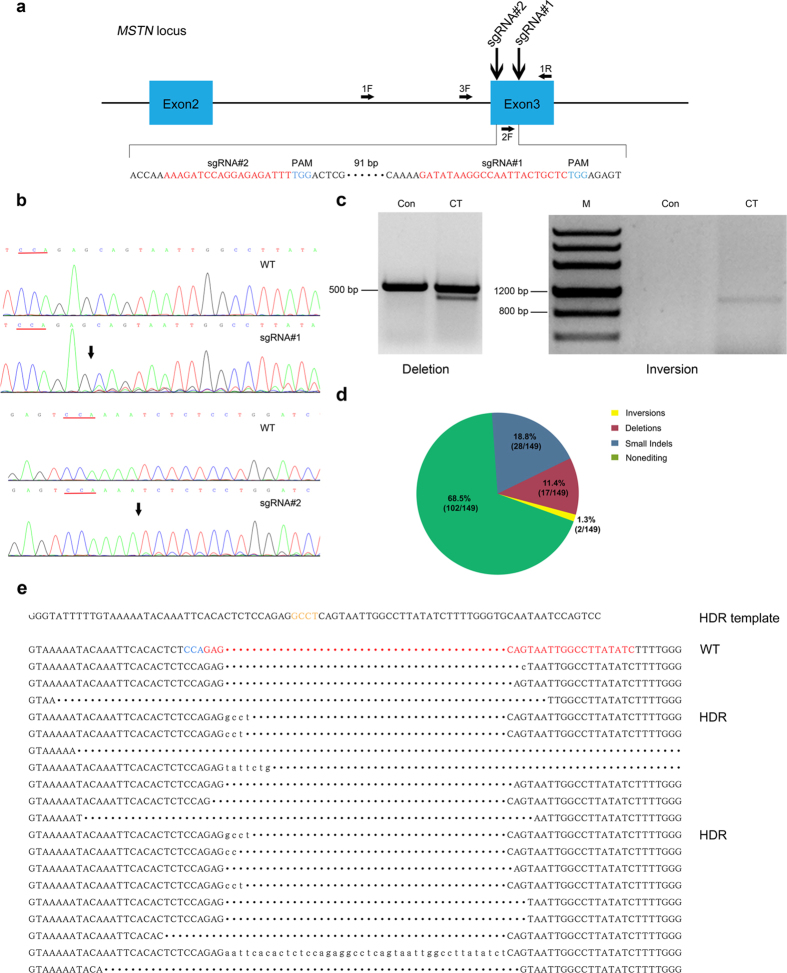
Editing of the porcine *MSTN* locus using the CRISPR/Cas9 system. (**a**) Schematic representation of sgRNAs specific to exon 3 of the porcine MSTN locus. SgRNA targeting sequences are highlighted in red, and PAMs are highlighted in blue. (**b**) Genomic sequences of CRISPR target regions in wild-type PFFs and transfected PFFs, as indicated. PAMs are underlined in red and the cleavage sites are labeled with arrow. (**c**) Dual sgRNA-induced mutation at the porcine MSTN locus. PCR results for the targeted regions from co-transfected (CT) and un-transfected (Con) PFFs are shown. Both deletion and inversion bands were detected using specific primers. (**d**) The dual sgRNA-induced deletion and inversion frequency were further determined based on 149 amplicons covering the target sites. (**e**). Sequence of the ssDNA donor (upper panel). The newly added nucleotides are highlighted in orange. Sequencing of sgRNA#1 and the ssDNA donor induced mutations in the MSTN gene (lower panel). PCR amplicons spanning the target sites were analyzed for indels. The wild-type sequences are placed on the first line, while mutant TA clones are placed below. Target sites are indicated in red, PAM in blue, and inserted or substituted bases are presented in lower case. Clones showing correct HDR are labeled to the right of the sequences.

The Belgian Blue and Piedmontese cattle breeds, which exhibit the double-muscled phenotype, harbor natural mutations in *MSTN* exon 3^13^. Therefore, we designed two porcine *MSTN*-specific sgRNAs, designated #1 and #2, to target the exon 3 region, expecting the resultant transgenic pigs to exhibit a similar phenotype to the DM cattle breeds (Fig. 2a). To assess the targeting efficiency of sgRNAs, *MSTN*-specific sgRNA-Cas9 encoding vectors were transfected into PFFs via electroporation as described previously. After 4 days of culture, the cells were collected, and PCR products covering the target site were examined through Sanger sequencing. There were obvious multi-peaks around the Cas9 cleavage site in the chromatogram, suggesting that *MSTN* mutations had been induced (Fig. 2b). The PCR amplicons were TA cloned and sequenced to further determine the mutation efficiency. The mutation efficiency of sgRNA#1 and sgRNA#2 were 21.79% and 12.5%, respectively (Table 1). Because both sgRNAs presented the desired targeting efficiency, we sought to explore dual sgRNAs mediated gene targeting at the *MSTN* locus. The two sgRNA-Cas9 encoding vectors were co-transfected into PFFs, and related regions were amplified. Indeed, we observed an additional smaller band and a specific inversion band compared with the untreated control (Fig. 2c).The TA cloning results also confirmed that deletions and inversions had occurred between the two sgRNA targeting sites (Fig. 2d). The mutation efficiency induced by the dual sgRNAs was quite high (approximately 31.5%) compared with that of individual sgRNAs.Because ssDNA-mediated homology-directed repair has been reported at ZFN- or TALEN-induced DSBs^23^,^24^,^25^, we next sought to explore whether our sgRNA-cas9 encoding vectors could achieve ssDNA-mediated homologous recombination at the porcine *MSTN* locus. An 85 bp antisense oligonucleotide that inserts 4 bp at the sgRNA#1 cleavage site was synthesized and then co-transfected into PFFs with the sgRNA#1-cas9 targeting vector. Four days later, the PCR amplicons encompassing the desired site were sequenced and TA cloned. Both the chromatogram of the mixed PCR amplicons and sequencing results for the TA clones showed the intended homologous recombination, although the efficiency was quite low (Fig. 2e, Table 1 and Supplementary Fig. 2).

### Generation and identification of MSTN KO Pigs

**Figure 3:**
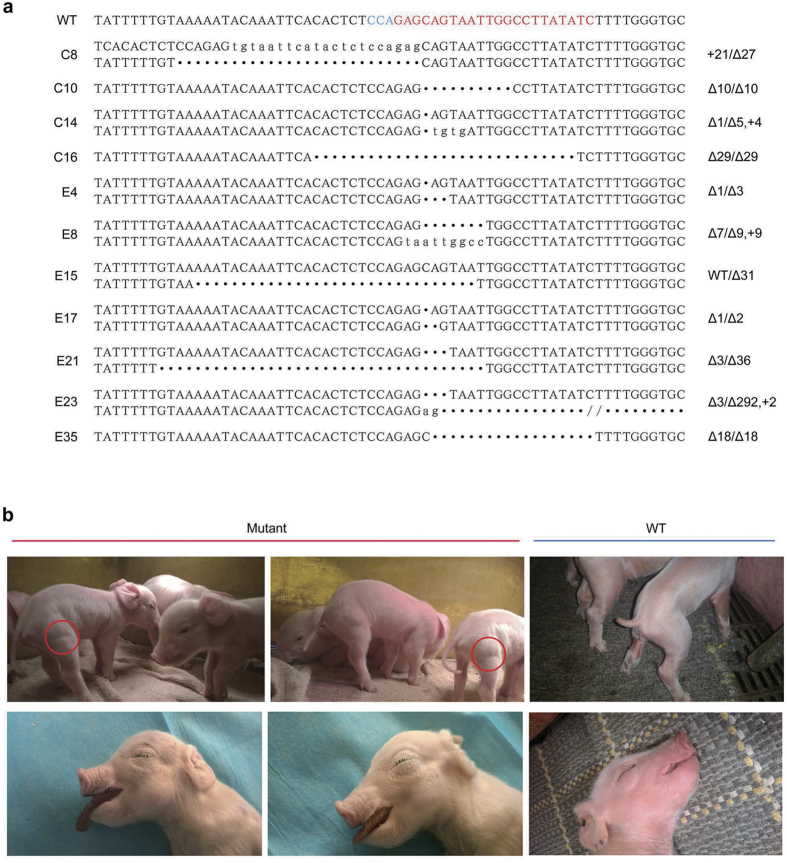
Generation of gene-targeted piglets via SCNT. (**a**) Genotypes of a portion of the *MSTN* mutant single-cell colonies. The target sites are labeled in red and PAM in blue. C8,C10,C14 and C18 are Landrace PFFs while E4,E8,E15,E17,E21,E23 and E35 are Erhualian PFFs. (**b**) Newborn piglets harboring MSTN mutations and some of the piglets show visible intermuscular boundaries (upper panel) and enlarged tongues (lower panel) compared with the WT controls.

SgRNA#1-Cas9 was electroporated into fetal fibroblasts from Landrace and Erhualian (China) pigs as described above. After 4 days of culture, the cells were trypsinized and plated at the proper density to enable the generation of single-cell colonies. After approximately 8 days of culture, single-cell colonies were picked and propagated for further analysis. The PCR products spanning the target sites in the colonies were sequenced directly. Among the Landrace PFFs, 51 colonies were examined, and mutations were found in 10 of the colonies. Among the Erhualian PFFs, 37 colonies were sequenced, and mutations were identified in 13 of the colonies (Supplementary Fig. 3). The PCR amplicons from some of the single-cell colonies with double peaks were TA cloned and sequenced to determine the exact mutation sequences (Fig. 3a).

**Figure 4:**
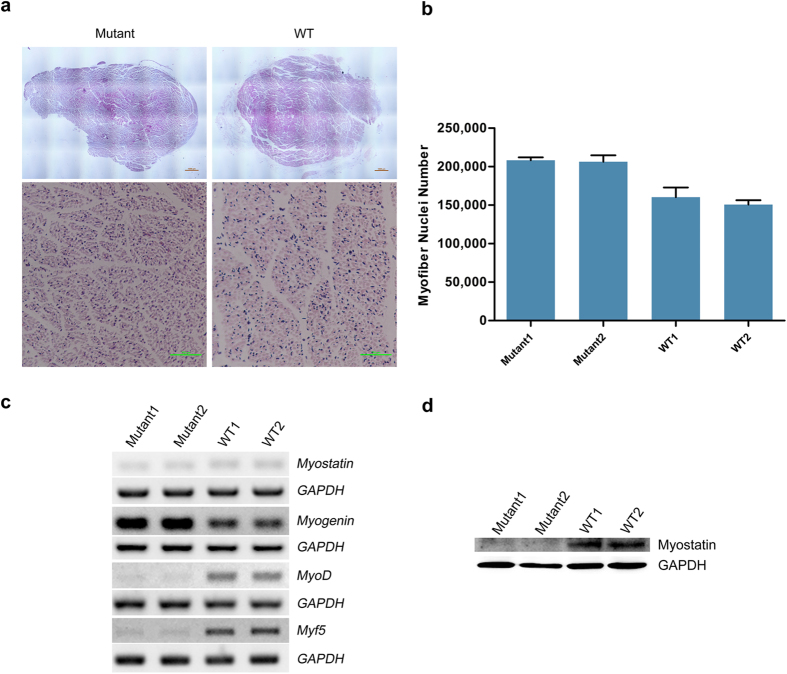
Identification of MSTN biallelic mutant piglets. (**a**) Representative images of H&E-stained sections to compare the total longissimus dorsi muscle cross-sectional area at a × 4 magnification and general morphology at a × 20 magnification. (**b**) Quantification of number of myofiber nuclei in longissimus dorsi muscle slices of two mutant and two WT piglets. n = 5. Each bar indicates the mean ± S.E.M. (**c**) Semi-quantitative RT-PCR analysis of MSTN and myogenic regulatory factors in mutant and WT pigs. (**d**) Western blotting analysis of MSTN levels in mutant and WT piglets.

Four *MSTN* KO Landrace fetal fibroblast cell colonies (cells C8, C10, C14, and C16) were chosen as donor cells for SCNT (Table 2). Specifically, 955 reconstructed embryos derived from Landrace *MSTN* KO cells were transferred to five estrous surrogates. Among these surrogate mothers, four carried to term and gave birth to 12 cloned piglets (Fig. 3b). DNA sequencing analysis results revealed that 8 of the Landrace newborns carried the expected mutations at the target loci, but they all died within a week (Table 3). There were four unexpected WT pigs due to impure colonies that contained a few wild-type cells. To detect the integration of CRISPR plasmids, we amplified the Cas9 domain, and no integration was found in any of the mutant piglets (Supplementary Fig. 4). The average birth weight of the mutant piglets was 15% larger than their WT littermates.Some of the knockout piglets showed obvious intermuscular boundaries and remarkably enlarged tongues compared with the wild-type controls (Fig. 3b). Moreover, histological analysis of slices of the longissimus dorsi revealed that some of the mutant piglets exhibited more myofiber nuclei compared with the WT group, which indicates that more myofibers existed in the mutant group (Fig. 4a,b). We then examined the mRNA levels of *MSTN* and related signaling pathway factors via RT-PCR. Only slight decrease in the *MSTN* mRNA level was observed in the fibroblasts of the mutant pigs (Fig. 4c). The RT-PCR results also showed that the *Myogenin* mRNA level was higher in the fibroblasts of the mutant pigs than the WT controls, whereas the levels of *Myf5* and *MyoD* mRNA were lower in the mutant pigs than the WT pigs (Fig. 4c). Finally, we performed western blotting and found that the level of the MSTN precursor was lower in the heart muscle of mutant pigs compared with the heart muscle of the wild-type controls (Fig. 4d). These results suggest that mutations altered the expression of *MSTN* and members of the related signaling pathway, which may account for the phenotype of the mutant cloned pigs. A more detailed analysis of the DM phenotype will be performed in our subsequent research.

### Off-target analysis of the cloned pigs

**Figure 5:**
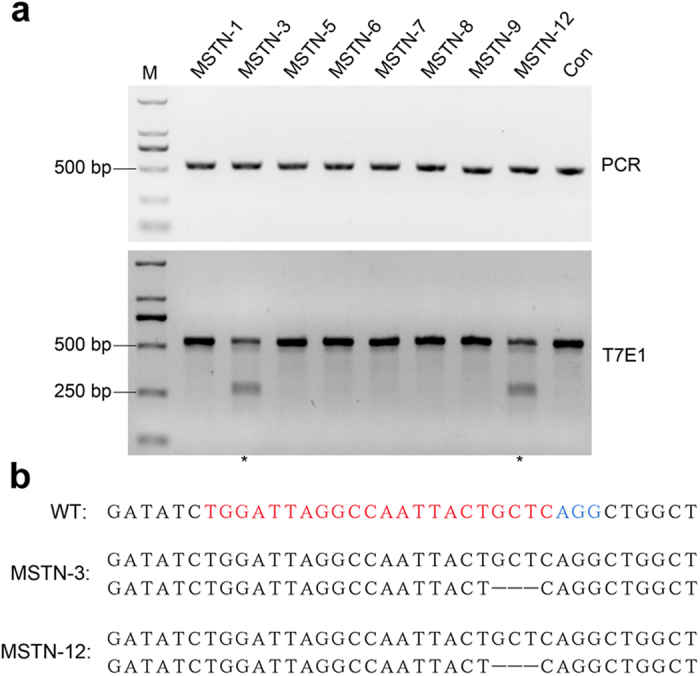
Detection of sgRNA#1-mediated off-target effects in mutant pigs. (**a**) PCR products covering potential off-target site 16 were subjected to T7 endonuclease I (T7E1) assays. Two pigs (asterisk) showed cleavage bands indicating non-specific mutations in OTS-16. (**b**) Further cloning of the PCR products confirmed the mutations. The target sites and PAM of OTS-16 are labeled in red and blue, respectively.

To assess the off-targeting effect of the CRISPR/Cas9 system in the *MSTN* KO pigs, we screened the pig genome and predicted 24 potential off-target sites (OTS) using BLAST (Supplementary Table 2). The genomic DNA of the mutant cloned pigs was analyzed through DNA sequencing and the T7E1 assay using primer pairs that cover potential off-target loci(Supplementary Table 3). Only one potential OTS (OTS-16) in two knockout piglets derived from the same cell colony was found to be mutated (Fig. 5), suggesting that sgRNA#1 exhibited a high level of specificity in the PFFs.

## Discussion

The transfection efficiency of donor cells has always been a key factor affecting the generation of genetically modified large animals, especially those in which a precise modification is achieved via homology-directed repair. A low transfection efficiency results in fewer modified cells, making it more time-consuming and costly to obtain the desired cell colonies. In this study, we found that Opti-MEM can be used as an effective transfection medium, thereby simplifying the transfection process. We also showed that our modified electroporation protocol improved the transfection efficiency of PFFs to nearly 90% and dramatically enhanced the efficiency of gene targeting.

Furthermore, we demonstrated the feasibility of Cas9/gRNA-mediated gene editing at the *MSTN* locus of porcine fetal fibroblasts. The mutation efficiency of a single Cas9/gRNA was up to 21.79% according to the TA cloning results. Similar to studies using ZFN^26^ and TALEN^27^, we demonstrated that co-transfection of two Cas9/gRNAs targeting the *MSTN* gene could induce long fragment deletions and inversions, which would be helpful for clarifying the function of certain domains. Moreover, our results suggest that dual sgRNAs are more efficient in inducing mutations than individual sgRNAs, which is consistent with previous studies^28^,^29^. Interestingly, the frequency of simultaneous cleavage by two sgRNAs was higher than expected. It is possible that this enhanced efficiency was partly due to homology-directed repair in which one mutant allele served as the template for the other allele^30^. Alternatively, the DSBs induced by the first sgRNA may have increased the genomic accessibility of a nearby region, which could facilitate the cleavage of the second sgRNA. This strategy could be used to target intractable genes. Livestock breeds bearing natural mutations are precious resources for animal breeding, but they are rare in nature, and such mutations occur randomly. Using traditional gene targeting technology to introduce mutations is inefficient and time-consuming. Hence, achieving precise and efficient modification at targeted sites would vigorously promote the development of animal breeding. It has been reported that the frequency of homology-directed repair can be greatly increased in the presence of DSBs^31^. Several studies have achieved precise ssDNA-mediated modification using ZFN, TALEN and CRISPR/Cas9^10^,^23^,^25^. In this study, we also demonstrate that ssDNA can induce homology-directed repair in combination with CRISPR/ Cas9. The efficiency observed in our study is similar to a previous study^24^. Given the similar DSB efficiencies, it appears that it is more difficult to achieve HDR at the *MSTN* locus compared with other genes. Of note, it has been reported very recently that suppression of key NHEJ pathway proteins greatly increases the efficiency of HDR in mammalian cells^32^,^33^. Perhaps these new approaches can be applied to the porcine *MSTN* locus to achieve more effective gene editing. Thorough research into the mechanism of DNA repair would help clarify these differences. Interestingly, the mutation efficiency of sgRNA#1 appeared to increase when co-transfected with the ssDNA template. This finding is consistent with a recent study on mouse embryonic stem cells^34^. However, additional trials need to be performed to investigate whether the ssDNA template affects the mutation efficiency of Cas9/gRNA.

Recently, the surveyor assay and T7 E1 assay have been widely used to detect the mutation efficiency induced by custom endonucleases such as ZFN, TALEN and CRISPR/Cas9. Both methods largely depend on randomness and efficiency during re-annealing. On the other hand, several studies have specifically chosen restriction enzyme sites as targets to facilitate the detection of mutations. We believe that this compromises the flexibility of custom endonucleases and is unnecessary. In this study, we preliminarily evaluated the mutation efficiency by observing the chromatogram of PCR amplicons, and the results were consistent with a subsequent TA cloning experiment. Therefore, it is convenient to roughly estimate the mutation efficiency by analyzing the height of multi-peaks in the chromatogram.

Direct editing of zygotes often results in chimeric founder animals, and the mutations are unpredictable, whereas somatic cell modification followed by cloning with drug selection increases the risk of transgenic animal products^35^. By combining Cas9/gRNA with limited dilution, we acquired marker-free *MSTN* knockout single cell colonies. After SCNT, eight knockout piglets were born. Semi-quantitative RT-PCR and western blotting analysis demonstrated that the expression of *MSTN* and related signaling pathway factors was altered in the biallelic mutant pigs. As expected, the mutant piglets presented some characteristics of the DM phenotype, such as enlarged tongues and clearly visible intermuscular boundaries^16^,^36^. Histological examination also showed that more myofiber nuclei existed in the longissimus dorsi of newborn *MSTN* knockout piglets, which suggests that the number of muscle fibers in pigs is regulated by MSTN during embryonic development. It has been well established that the alternation of muscle size is accompanied with the changes in the number of myofiber nuclei in order to maintain a constant myonuclear domain^37^,^38^. In this regard, there exists the possibility that the mutant piglets could exhibit larger muscle mass during postnatal development.The influence of MSTN on muscle hypertrophy and hyperplasia is complex. *MSTN*-null mice exhibit a 2–3 times higher muscle mass than wild-type animals, and the increase in mass results from a combination of muscle fiber hyperplasia and hypertrophy^12^. In contrast, double-muscled cattle display only a 20–25% increase in muscle mass, and the increased muscle weight appears to be the result of muscle fiber hyperplasia, rather than hypertrophy^13^,^39^,^40^. One possible explanation for this difference is that livestock animals such as pig and cattle may exhibit a decreased ability to reach the maximal limit of muscle size after generations of selective breeding for a higher lean meat percentage^13^. In this regard, it is possible that porcine *MSTN* mutations could only cause modest increase in muscle mass. Indeed, we found that the *MSTN* mutant piglets exhibit 15% increase in birth weight compared with the WT littermates, which is more consistent with the double-muscled cattle. These results demonstrate that CRISPR/Cas9-induced mutations can disrupt the function of MSTN and, thus, hold great promise for generating pigs with a greater muscle mass in the future.

In conclusion, we improved the transfection efficiency of PFFs to facilitate Cas9/gRNA gene targeting in the porcine genome. By inducing NHEJ, long fragment deletion/inversion and homology-directed repair, CRISPR/Cas9 can be used to generate genetically modified pigs with a high efficiency. To the best of our knowledge, this is the first evidence of *MSTN* biallelic mutant pigs obtained without a selection marker gene using the CRISPR/Cas9 system. The gene editing system employed in our study offers a convenient and safe way to generate genetically modified large animals without using a selection marker gene.

## Methods

### Ethics statement

All animal studies were approved by the Animal Welfare and Research Ethics Committee at Jilin University (Approval ID: 20140310), and all procedures were conducted strictly in accordance with the Guide for the Care and Use of Laboratory Animals.All surgery was performed under anesthesia, and every effort was made to minimize animal suffering.

### Plasmids and oligonucleotide

The vector backbone consisting of U6-sgRNA and Cas9 expression elements (42230) was purchased from Addgene. Targeting sgRNAs were designed with one G ahead. Two complementary sgRNA oligo DNAs were synthesized and then annealed to a double-strand DNA in the presence of 10 × NEB standard *Taq* buffer and ligated to the *BbsI* sites of the vector backbone to form the intact targeting plasmid. These constructs were confirmed through sequence analysis. The 85-bp oligonucleotides were synthesized and purified through PAGE (GENEWIZ, Suzhou, China).

### Preparation and culture of PFFs

The procedure for the isolation of PFFs used in our laboratory has been described previously^19^. Landrace and Erhualian (China) fetuses were obtained via hysterectomy of a pregnant (day 33) sow and washed in Dulbecco PBS (DPBS) (Gibco, Grand Island, New York, USA). Heads, tails, limbs and viscera were removed, and the remaining tissues were washed again in DPBS and cut into cubes of 1 mm^3^. Next, these tissues cubes were digested in cell culture medium containing 20% fetal bovine serum (FBS) (Gibco, Grand Island, New York, USA), 200 U/mL collagenase IV (type IV, 260 U/mg, Gibco), 25 U/mL DNase I(2,000 U/mg, Beyotime, Haimen, China) and 1% penicillin/streptomycin (Gibco) and cultured for 4–6 h at 39 °C under 5% CO2 in humidified air. Isolated PFFs were digested from the culture dishes using 0.1% (w/v) trypsin in DPBS and centrifuged at 500 × *g* for 5 min. After removal of the supernatant, PFFs were resuspended and cultured in new 10-cm cell culture dishes. Cells at passage 3 were frozen in FBS containing 10% dimethylsulfoxide. In the following experiments, cells were thawed and cultured in DMEM with 10% FBS at 39 °C under 5% CO_2_ in humidified air.

### Transfection and selection of PFFs

One day before transfection, PFFs were thawed and cultured in 10 cm dishes until reaching sub-confluence. Approximately 3 × 10^6^ PFFs in 250 μL of Opti-MEM (Gibco, Grand Island, New York, USA) were electroporated using BTX ECM 2001 in 2 mm gap cuvettes. The parameters for electroporation were as follows: 300 V, 1 ms, 3 pulses for 1 repeat. In different electroporation experiments, 25 μg of the corresponding plasmids (with or without 3 μM oligonucleotides) was used. PFFs were also transfected with pEGFP-N1 using FuGENE HD (Promega, Madison, Wisconsin, USA) according to the manufacturer’s protocol. After 36 h of recovery, the cells electroporated with sgRNA#1-Cas9 were plated into 10 dishes of 10 cm at an appropriate density. Individual cell colonies were picked and cultured in 24-well plates. After reaching 80%–90% confluence, the cell colonies were subcultured, and 10% of each colony was individually lysed using 10 μL of lysis buffer (0.45% NP-40 plus 0.6% Proteinase K) for 60 min at 56 °C and then 10 min at 95 °C. The lysate was used as a template for PCR. The primers were designed to cover the cleavage site. The 3F forward primer was 5′-GGCGAAGACCTCAGGGAAATTTATATTG-3′, and the 1R reverse primer was 5′-ACAGCGATCTACTACCATGGCTGGAATT-3′. The PCR conditions were 94 °C for 5 min; 94 °C for 15 sec, 62 °C for 10 sec, 68 °C for 15 sec, for 35 cycles; 68 °C for 8 min; hold at 16 °C. The PCR products were sequenced to detect mutations. Some PCR products were selected for ligation into a pLB vector (Tiangen, Beijing, China) and sequenced to determine the exact mutation sequences. The positive cell colonies were expanded and then cryopreserved. These cells were thawed and cultured to reach sub-confluence in a 24-well plate before SCNT. The primers 3F and 1R were also used to detect dual sgRNA-induced deletions, while the primers 1F (5′ CTTAAATACCAATAATTACCAGGTCT 3′) and 2F (5′ TACCCTCTAACTGTGGATTTTGAAGC 3′) were used to detect inversions.

### Fluorescence microscopy and flow cytometric analysis

PFFs were electroporated with pEGFP-N1 as described previously. The cells were observed 24 h post-electroporation using fluorescence microscopy (Olympus BX51) under appropriate excitation filters. The harvested cells were washed twice, then resuspended with 300 μL of PBS and analyzed using a BD Accuri C6 flow cytometer.

### SCNT and genotyping of mutant piglets

Positive colonies identified as gene knockouts via sequencing were selected as donor cells to perform pig SCNT, which was carried out as described by Lai^41^,^42^.

Genomic DNA was extracted from the tails of cloned piglets and analyzed via PCR using the 3F/1R primers. Some PCR products were selected for ligation into the pLB vector and sequenced to determine the exact sequences of both alleles.

To investigate whether integration of CRISPR plasmids occurred in the mutant piglets, the genomic DNA of these pigs was PCR amplified using the primers 5′-TGCCACCATGGACTATAAGGAC-3′ and 5′-TCTTTCTCAGGTGGTAGATGGTG-3′ for Cas9 detection. The PCR conditions were 94 °C for 5 min; 94 °C for 30 sec, 62 °C for 30 sec, 72 °C for 30 sec, for 35 cycles; 72 °C for 8 min; hold at 16 °C.

### Morphological analysis of muscles

The longissimus dorsi muscles of two mutant piglets and two control piglets of the same age were collected for histological examination. The muscle samples were cross-sectioned at the widest part, and placed on glass slides. The slices were then stained with hematoxylin-eosin and analyzed through microscopy (Nikon, Tokyo, Japan). The cross-section area (CSA) and number of cell nuclei in each field of view were analyzed using the Image Pro Plus software (Media Cybernetics, Silver Spring, USA). Counts of myofiber nuclei were performed across five randomly selected fields of view in each slide, and the average number was calculated. Five independent counting experiments were performed for each muscle sample. Finally, the relative difference in the number of myofiber nuclei between the mutant and WT groups was determined based on the muscle cross-sectional area and density of nuclei (number of nuclei per field of view).

### Semi-quantitative RT-PCR

Fibroblasts were isolated from the tails of the mutant and control piglets at day 1. Total RNA was extracted using the TRIzol reagent (Tiangen, Beijing, China), and the RNA concentration was quantified using a NanoDrop spectrophotometer. First-strand cDNAs were generated through reverse transcription using 650 ng of total RNA and oligo-dT primers. The PCR products were analyzed via electrophoresis in agarose gels.The following primers were used: *GAPDH* (sense: 5′-GGTCTACATGTTCCAGTATGATTCC-3′; antisense: 5′-GATGGCATGGAC

CGTGGTCATGAGT-3′), MSTN (5′-AAAGGCCCAACTGTGGATATATCTG-3′; antisense: 5′-TG

ACCATTCTCATCTAAAGCTTTGA-3′), *Myf5*: (5′-TCAGACGAGGAAGAGCACGTGCGA-3′;

antisense: 5′-TCCGATCCACTATGCTGGATAAGC-3′), *Myogenin*: (5′-AGGCTACGAGCGGA

CTGAG-3′; antisense: 5′-CAGACACGGACTTCCTCTTACAC-3′), *MyoD*: (5′-AGTCAGGAGG

GACAGGATAGAGC-3′; antisense: 5′-CGAAACACGGGTCATCATAGAAG-3′).

### Western blotting analysis

Frozen heart muscle samples were ground in liquid N, and the resultant powder was solubilized in lysis buffer. After incubation for 1 h on ice, the extracts were centrifuged at 15,000 × g for 10 min. The resultant protein concentrations were determined using a BCA protein assay kit (Beyotime, Haimen, China). Equal amounts of proteins were separated through SDS polyacrylamide gel electrophoresis (SDS-PAGE) on a 12% separating gel, and the protein bands were electrophoretically transferred to nitrocellulose membranes. The membranes were then blocked for 2 h in 5% milk and TBST buffer at room temperature. The membranes were subsequently incubated overnight at 4 °C with a primary polyclonal antibody directed against mstn (GDF-8 [C-20]: sc-6884,Santa Cruz Biotech., Santa Cruz, CA) diluted 1:200 with TBST buffer. The membranes were subsequently washed 3 times for 10 min with TBST buffer, followed by incubation for 1 h with a horseradish peroxidase-labeled anti-goat secondary antibody diluted 1:1,000 with TBST buffer. The proteins were visualized with the ECL-Plus western blotting reagent.

### Off-target analysis

All potential OTS that showed homology to sgRNA#1 plus protospacer adjacent motifs (PAMs) were predicted by scanning the porcine genome using BLAST. The genomic DNA of the mutant piglets was analyzed via PCR and DNA sequencing to determine off-target effects. These primer sequences are listed in Supplementary Table 2. For OTS-16, the T7E1 assay was performed according to the manufacturer’s protocol. Briefly, PCR products surrounding OTS-16 in the MSTN knockout pigs were purified and digested with T7E1 for 30 min at 37 °C. All digested products were then analyzed through electrophoresis in 2% agarose gels.

## Additional Information

**How to cite this article**: Wang, K. *et al.* Efficient Generation of Myostatin Mutations in Pigs Using the CRISPR/Cas9 System. *Sci. Rep.* **5**, 16623; doi: 10.1038/srep16623 (2015).

## Supplementary Material

Supplementary Information

## Figures and Tables

**Table 1:** Comparison of the mutation efficiency induced by sgRNA alone or sgRNA + HDR template.

Cell	Targeting vector	HDR template	Mutation	TA colonies	Mutation efficiency	HDR efficiency (%)
Erhualian PFFs	sgRNA#1	–	17	78	21.79%	–
Erhualian PFFs	sgRNA#2	–	10	80	12.50%	–
Erhualian PFFs	sgRNA#1	ssDNA-85	19	55	34.55%	2/55(3.64%)

**Table 2:** SCNT results for the generation of gene-targeted pigs.

Target gene	Cell pool	Transferred embryos	No. recipients	No. (%) pregnancies	No. born	No. (%) mutants
**MSTN**	C10,C14,C16	195	1	1	2	1
	C8,C10,C14,C16	320	2	2	7	6
	C8, C10, C16	440	2	1	3	1
**Total**		955	5	4 (80%)	12	8 (67%)

**Table 3:** Summary of knockout piglets for their health status and genotypes.

Piglet ID	Gestation period (days)	Birth weight (g)	Health status	Donor cell
**MSTN-1**	117	700	Died at day 4	C14
**MSTN-3**	117	1240	Died at day 2	C8
**MSTN-5**	117	570	Died at day 1	C14
**MSTN-6**	117	1020	Stillborn	C10
**MSTN-7**	117	880	Died at day 4	C10
**MSTN-8**	117	1430	Died at day 3	C16
**MSTN-9**	117	490	Died at day 1	C10
**MSTN-12**	116	710	Stillborn	C8
