# Supplementary material for: Efficient Generation of Myostatin Mutations in Pigs Using the CRISPR/Cas9 System
Source: Sci Rep. 2015 Nov 13;5:16623. doi: 10.1038/srep16623 (PMC4643223; doi:10.1038/srep16623)
Supplement: Supplementary Information [file srep16623-s1.doc]

**Efficient Generation of Myostatin Mutations in Pigs Using the CRISPR/Cas9 System**

Kankan Wang^1,&^, Hongsheng Ouyang^1,&^, Zicong Xie^1^, Chaogang Yao^1^, Nannan Guo^1^, Mengjing Li^1^, Huping Jiao^1^,**^*^**, Daxin Pang^1^,**^*^**.

^1^Jilin Provincial Key Laboratory of Animal Embryo Engineering, College of Animal Sciences, Jilin University, Changchun, Jilin Province,People’s Republic of China

^&^These authors contributed equally to this work.

***** Corresponding author

D. Pang and H. Jiao, College of Animal Sciences, Jilin University, 5333 Xian Road, Lvyuan District, Changchun 130062, Jilin Province,People’s Republic of China

E-mails: [jiaohp@jlu.edu.cn](mailto:jiaohp@jlu.edu.cn)(H. Jiao); [pdx@jlu.edu.cn](mailto:pdx@jlu.edu.cn)(D. Pang);

**Supplementary Figures**

**
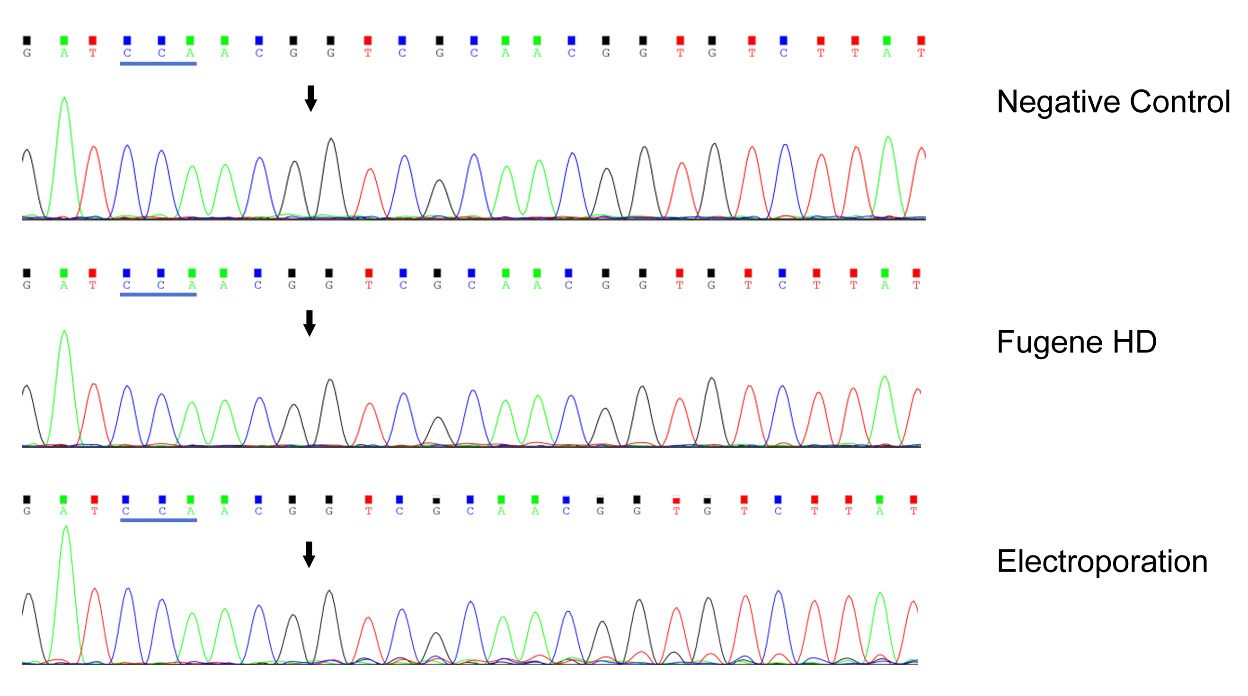
**

**Supplementary Figure 1**.Optimized electroporation parameters dramatically enhanced the targeting efficiency of CRISPR/Cas9. The *EIF4G1*-specific sgRNA-Cas9 vector were transfected into PFFs viaindicated methods. Sequencing chromatograms of PCR products encompassing the target site are shown. The electroporation group had more obvious multi-peaks around the targeting sites compared with the FuGENE HD group. The cleavage sites are labeled with arrow and PAMs with underline.

**Supplementary Figure 2.**Sequencing of PCR amplicons from PFFs transfected with sgRNA#1-Cas9 targeting vector and the ssDNA-85 donor. The cleavage sites are labeled with arrow.

C5:

C7:

C8:

C10:

C14:

C16:

C18:

C21:

C23:

C51:

E4:

E7:

E8:

E9:

E15:

E16:

E17:

E21:

E23:

E25:

E32:

E35:

E37:

**Supplementary Figure 3.** Sequencing chromatogram of PCR products harboring targeting sites from mutant single-cell colonies. C5,C7,C8,C10,C14,C16,C18,C21,C23 and C51 are Landrace PFFs while E4,E7,E8,E9,E15,E16,E17,E21,E23,E25,E32,E35 and E37 are Erhualian PFFs.

**
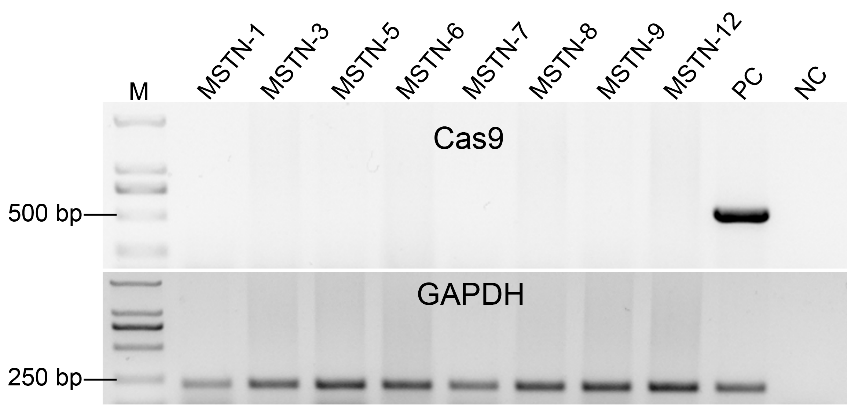
**

**Supplementary Figure 4.**PCR amplification of Cas9 confirmed no integration of CRISPR plasmid in mutant cloned pigs. GAPDH was used to validate the quality of the genome samples. PC represents the positive control, and NC represents the negative control.

**Supplementary Tables**

**Supplementary Table 1**Influence of different transfection methods on sgRNA induced mutations at porcine *EIF4G1* locus.

| **Transfection methods** | **Mutations** | **TA colonies** | **Mutation efficiency** |
| --- | --- | --- | --- |
| **FuGENE HD** | 0 | 28 | 0% |
| **Electroporation** | 8 | 33 | 24.24% |

.

**Supplementary Table 2 List of putative off-target sites homologous to sgRNA#1.PAM sequences are labeled in blue. Base substitutions are shown in red**

| **sgRNA#1** | **G** | **A** | **T** | **A** | **T** | **A** | **A** | **G** | **G** | **C** | **C** | **A** | **A** | **T** | **T** | **A** | **C** | **T** | **G** | **C** | **T** | **C** | **T** | **G** | **G** |
| --- | --- | --- | --- | --- | --- | --- | --- | --- | --- | --- | --- | --- | --- | --- | --- | --- | --- | --- | --- | --- | --- | --- | --- | --- | --- |
| **OTS1** | G | A | A | A | A | A | T | G | G | A | T | A | A | T | T | A | C | T | G | C | T | C | T | G | G |
| **OTS2** | G | G | A | T | C | C | T | G | T | A | T | A | A | T | T | A | C | T | G | C | T | C | T | G | G |
| **OTS3** | T | T | C | T | C | A | A | G | G | C | C | A | A | A | T | A | C | T | G | C | T | C | T | G | G |
| **OTS4** | T | C | A | G | G | A | A | G | G | C | C | A | A | G | T | A | C | T | G | C | T | C | A | G | G |
| **OTS5** | G | G | T | G | T | T | C | A | A | A | C | A | A | T | T | A | C | T | G | C | T | C | A | G | G |
| **OTS6** | A | A | A | T | G | T | T | T | G | C | C | A | A | T | T | C | C | T | G | C | T | C | T | G | G |
| **OTS7** | T | T | G | G | A | A | C | A | G | C | C | A | C | T | T | A | C | T | G | C | T | C | T | G | G |
| **OTS8** | T | G | G | T | A | T | G | T | G | C | C | A | A | T | T | C | C | T | G | C | T | C | T | G | G |
| **OTS9** | G | G | T | G | T | T | C | A | A | A | C | A | A | T | T | A | C | T | G | C | T | C | A | G | G |
| **OTS10** | G | C | T | A | C | A | A | G | G | C | C | A | A | C | T | A | C | T | G | C | T | C | T | G | G |
| **OTS11** | G | A | C | T | G | G | G | C | C | G | G | A | A | T | T | A | C | T | G | C | T | C | C | G | G |
| **OTS12** | G | T | G | C | A | C | T | A | G | C | C | A | A | T | T | A | C | G | G | C | T | C | C | G | G |
| **OTS13** | G | T | T | T | A | G | A | G | C | C | A | A | A | T | T | A | C | T | G | C | T | C | G | G | G |
| **OTS14** | A | A | A | T | A | A | A | T | A | G | T | A | A | T | T | A | C | T | G | C | T | C | G | G | G |
| **OTS15** | T | A | A | T | T | T | C | T | G | A | G | A | A | T | T | A | C | T | G | C | T | C | A | G | G |
| **OTS16** | T | G | G | A | T | T | A | G | G | C | C | A | A | T | T | A | C | T | G | C | T | C | A | G | G |
| **OTS17** | T | G | C | A | A | C | T | A | G | T | C | A | A | T | T | A | C | T | G | C | T | C | A | G | G |
| **OTS18** | C | C | T | T | A | C | A | C | A | G | T | A | A | T | T | A | C | T | G | C | T | C | A | G | G |
| **OTS19** | T | G | A | C | T | T | G | C | T | C | C | A | A | T | T | A | C | T | G | C | T | C | A | G | G |
| **OTS20** | A | G | G | A | A | G | A | C | T | T | A | A | A | T | T | A | C | T | G | C | T | C | A | G | G |
| **OTS21** | G | C | T | T | T | C | A | T | A | G | T | A | A | T | T | A | C | T | G | C | T | C | A | G | G |
| **OTS22** | A | C | A | A | C | C | T | T | G | C | C | A | A | T | T | A | C | T | G | C | T | C | A | G | G |
| **OTS23** | C | A | A | T | A | A | C | A | G | C | C | A | A | T | T | A | C | T | G | C | C | C | A | G | G |
| **OTS24** | C | A | A | T | A | A | C | A | G | C | C | A | A | T | T | A | C | T | G | C | C | C | A | G | G |

**Supplementary Table 3** Primers for PCR amplification of off-target sites.

| Primers | Sequences (5' to 3') | Amplicon (bp) |
| --- | --- | --- |
| MSTN-OTS1 | GCCTAAGGCAACTGATCGTCCCT | 551 |
|  | GTATCTGTCTGGAGAATGAAGATTG |  |
| MSTN-OTS2 | TTCAGACATACACAAAAGTAGAGA | 516 |
|  | TTGCTGCAGAGATGCTATTGATCT |  |
| MSTN -OTS3 | ATTTTTCTCTGGGTGTGATGTGTG | 492 |
|  | TGTGTGCCAGTTTTGATACTTAGG |  |
| MSTN -OTS4 | ATAATCCCTTCCACTTATATTCGG | 515 |
|  | TTCCCTGCTGGTCTAGCAACTAAG |  |
| MSTN -OTS5 | GGTTTCACACAACATCCTGCCTCA | 506 |
|  | CATTTTCCCAACTTCGGTCACACG |  |
| MSTN -OTS6 | CCTCTTGCCACCCAGTGTTCCTAT | 521 |
|  | GTGGAACCTCTACAGGTCCATGGA |  |
| MSTN -OTS7 | CAATTTTGGATCATAGGACTCCAG | 482 |
|  | TCATCATCACTCTTATTAGACGAC |  |
| MSTN -OTS8 | GTTGCTGTGTTAGAGCAGCAGTGC | 520 |
|  | AATGCTGGAGCCTCCGTTGAAGAG |  |
| MSTN -OTS9 | ACATTTTCCCAACTTCGGTCACAC | 504 |
|  | TTCACACAACATCCTGCCTCAACA |  |
| MSTN -OTS10 | GAGAAATCAATAATCTCTGTTCCG | 495 |
|  | TTAGGAGCAGCCACAGCGATCCAC |  |
| MSTN – OTS11 | AGCAGGGGCTCAATAATGGGCGCT | 544 |
|  | CCTCCTCCTTGGTCTCCCGTTTCT |  |
| MSTN – OTS12 | CCAATTGGTTTTGGTGCCTTTTAA | 530 |
|  | AACGTCTTCTGTCTTCTCTGTTTT |  |
| MSTN – OTS13 | GTGAGCAAGTGTGAAGGTGAAGGA | 508 |
|  | ACGGAGGTAAACAAGGAGAGTAAA |  |
| MSTN – OTS14 | AAACCCAACTAGCATCCATGAAGA | 501 |
|  | CATAGAAGACAAATTTATGGCTAC |  |
| MSTN – OTS15 | TGAGTGCTTAGGAGTTATATAACC | 487 |
|  | ATAGAGAGTTGAATGGGGTAAAGG |  |
| MSTN – OTS16 | GGGACTTGGTTATGGATGTGCTTC | 532 |
|  | ACCACCTGCACTATCCCAAATGTT |  |
| MSTN – OTS17 | CCGAGGTGGGCATCTTAGTCTTAT | 556 |
|  | GCTCTGGCTTTGTATGTATGTTCT |  |
| MSTN – OTS18 | TCGAACCTGGTCAGATTCATTTCC | 556 |
|  | ATACCTGGAATAACTGGAGATACC |  |
| MSTN – OTS19 | CCATTCGGGTTTTCACTTTAAGTA | 566 |
|  | TGTGGACAAATAGTAGTAGATGCC |  |
| MSTN – OTS20 | GTCGAGTGAATGGACAAAGTAATG | 515 |
|  | TAGAAATCTGCCTTCTTGACTTAG |  |
| MSTN – OTS21 | TATGGAAGATAAGTTCAATAGTGG | 491 |
|  | AACTCAATATTACTTCAGATCCTC |  |
| MSTN – OTS22 | GCCTTGTACTGAAGTCCCATTTTA | 510 |
|  | TGTCCAGTTGTGCCTTTACTAGAT |  |
| MSTN – OTS23 | GCAGATGAAAGGTGTCAAATGCGG | 534 |
|  | GCCCAACTTTGCTGTGAGAGCGTC |  |
| MSTN – OTS24 | GTGAGAGCGTCTTCTGAAAGGACG | 468 |
|  | AGATGCTGAGAAACAGGCTGGCTG |  |
